# First Occurrence of *Platycladus* from the Upper Miocene of Southwest China and Its Phytogeographic Implications

**DOI:** 10.1371/journal.pone.0115141

**Published:** 2014-12-17

**Authors:** Jing-Yu Wu, Su-Ting Ding, Qi-Jia Li, Zhen-Rui Zhao, Bai-Nian Sun

**Affiliations:** 1 Key Laboratory of Mineral Resources in Western China (Gansu Province), School of Earth Sciences, and College of Earth and Environmental Sciences, Lanzhou University, Lanzhou, 730000, China; 2 State Key Laboratory of Palaeobiology and Stratigraphy, Nanjing Institute of Geology and Paleontology, CAS, Nanjing, 210008, China; Institute of Botany, China

## Abstract

*Platycladus* Spach is native to Central China, but its natural occurrences are very difficult to establish. According to molecular phylogenetic data, this genus might have originated since the Oligocene, but no fossil record has been reported. Here, we describe eight foliage branches from the upper Miocene in western Yunnan, Southwest China as a new species, *P. yunnanensis* sp. nov., which is characterized by foliage branches spread in flattened sprays, and leaves decussate, imbricate, scale-like and dimorphic. The leaves are amphistomatic, and the stomata are elliptical or oblong, haplocheilic, and monocyclic type. Based on a detailed comparison with the extant genera of Cupressaceae *sensu lato*, our fossils are classified into the genus *Platycladus*. The occurrence of *P. yunnanensis* sp. nov. indicates that this genus had a more southernly natural distribution in the late Miocene than at present. Molecular phylogeny and fossil records support a pre-Oligocene common ancestor for the genera *Platycladus*, *Microbiota* and *Calocedrus*. The separation of the three taxa was most likely caused by the arid belt across Central China during the Oligocene. In addition, the cooling down of the global temperature and the strengthening of Asian monsoon since the Miocene will further promote the migration of these genera.

## Introduction

*Platycladus* Spach, one of the 30 genera in the Cupressaceae *s.l.*, is a distinct genus of evergreen tree with one extant species, *P. orientalis* L., also known as Chinese arborvitae [1], [2]. *Platycladus orientalis* currently occurs in Central China [2], and was introduced to North Korea and the Russian Far East due to its adaptation of a wide range of climate and soil conditions [1], [3]. However, the natural distribution of *Platycladus* is difficult to distinguish owing to extensive cultivation and planting in the past [1], [2].

The molecular phylogenetic data indicate that *Platycladus* and *Microbiota* Komarov form a clade that is closely related to *Tetraclinis* Masters and *Calocedrus* Kurz [4]–[6]. The divergence of the *Platycladus*–*Microbiota* clade is considered to be ca. 33 Ma (early Oligocene) [4], [5]. The genus *Tetraclinis* has a wide historical distribution in western North America and Europe, but did not cross into Asia [7]. The floristic exchange of *Calocedrus* between eastern Asia and North America before the Oligocene via the Bering land bridge has been demonstrated [8], [9]. In contrast, the earliest fossil record of *Microbiota* can only be traced back to the Pliocene in Russia [10], and to date no fossil record of *Platycladus* has been reported. In the present study, we describe a new species as *Platycladus yunnanensis* sp. nov. from the upper Miocene in West Yunnan Province, Southwest China, based on a detailed comparison of gross morphology and cuticular features with the extant Cupressaceae. As the first record of *Platycladus,* the occurrence of the present fossil species will provide us evidence to recognize the natural distribution and migration of this conifer in the past.

## Materials and Methods

### Geological setting

**Figure 1:**
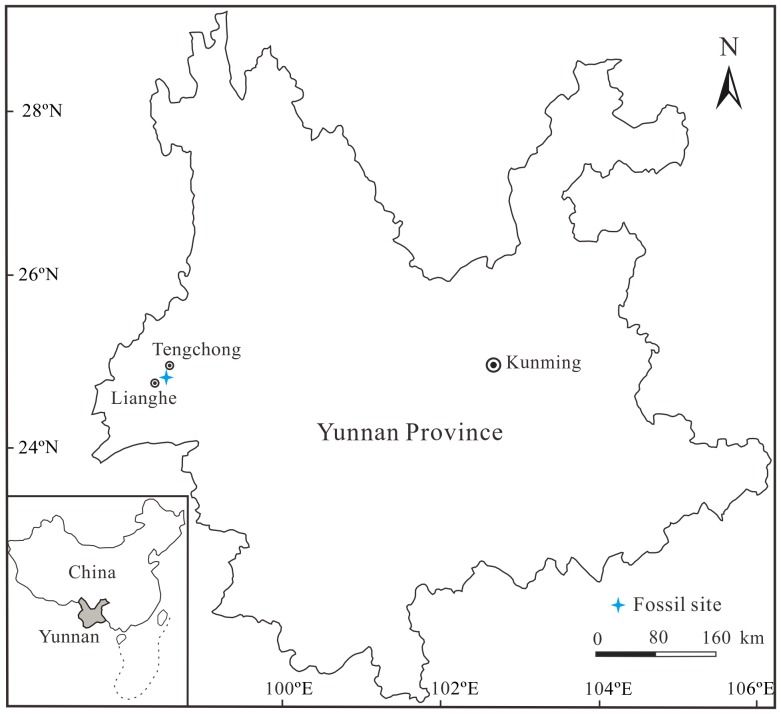
Simplified geological map of the fossil site in Yunnan Province, Southwest China.

**Figure 2:**
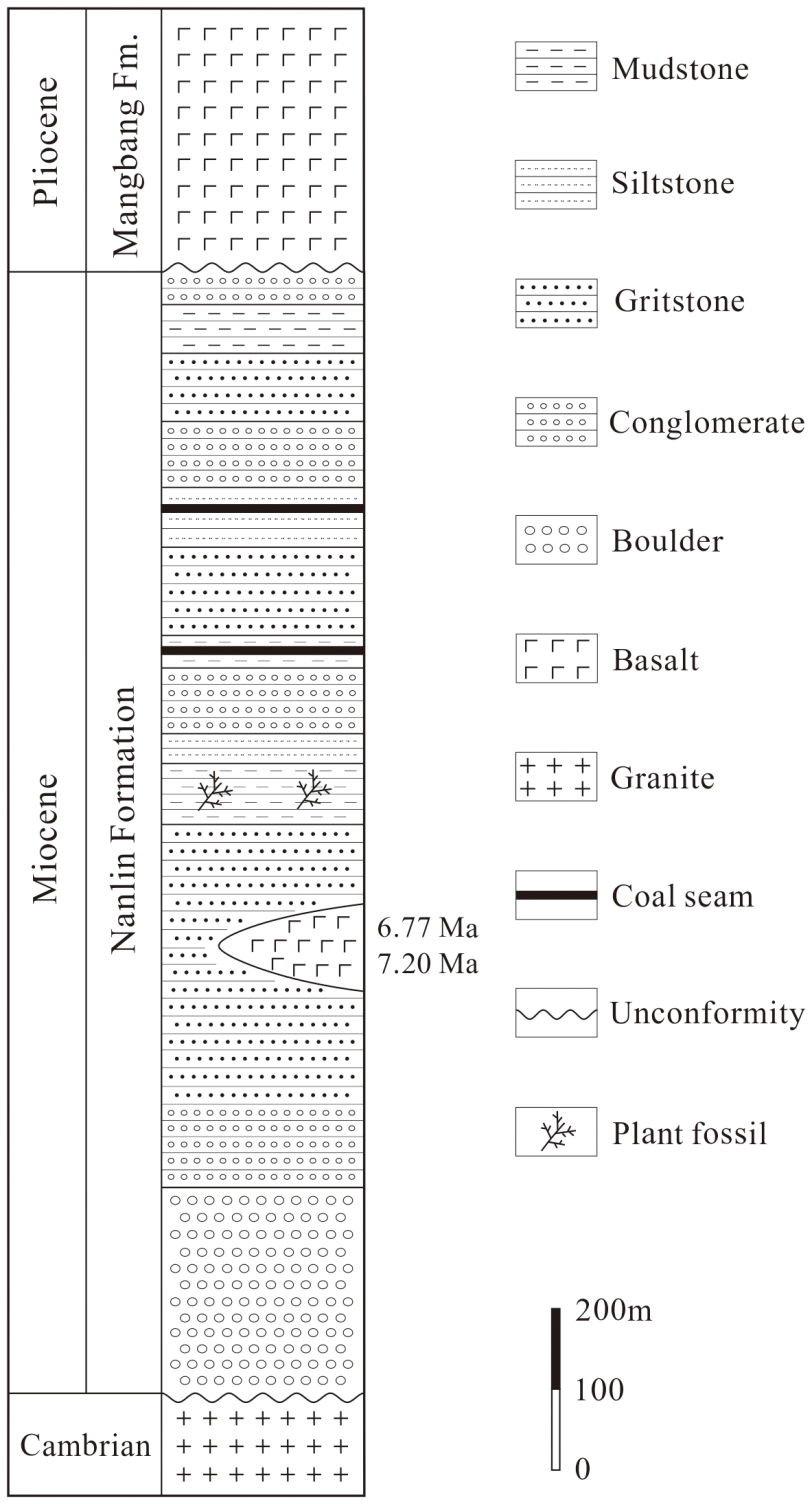
The stratigraphic section through the upper Miocene Nanlin Formation in Lianghe County, Yunnan Province, Southwest China [11]–[13].

The fossil branches studied here were collected from the Miocene Nanlin Formation at Nongbie Village (24°51'46"N, 98°24'58"E; Fig. 1), Lianghe County, Yunnan Province, China. The Nanlin Formation unconformably underlies the Pliocene Mangbang Formation and consists mainly of conglomerates, sandstones, siltstones, mudstones and basaltic rocks [11] (Fig. 2). The Nanlin Formation has been assigned to the Miocene according to the plant fossil assemblage [11], [12]. The basaltic rocks within the formation in Lianghe County were radiometrically dated at 7.20±0.22 Ma and 6.77±0.30 Ma using the K–Ar dating method [13]. Therefore, the fossiliferous layers studied here can be assigned to the late Miocene.

### Fossil material and preparation

After photographing with a SONY NEX–7 (SEL30M35), the leaf fragments were sampled from the fossil bearing matrix with a scalpel and placed in water. The fragments were immersed in 10% HCl overnight, washed and then immersed in 40% HF for 48 h. After washing in distilled water, the fragments were macerated with 45% HNO_3_ for 24 h. When the color of the fragments was changed from black to sandy beige, the samples were washed and then treated with 5% NH_4_OH for 5 min. The adaxial and abaxial cuticles were separated with a dissecting needle under a stereomicroscope. After staining with Safranin T, the cuticles were mounted on slides, embedded in glycerine jelly and sealed with nail polish, then photographed under a light microscope (Leica DM4000B). Unstained cuticles and unmacerated fragments were mounted on a stub and coated with gold, examined and photographed using a scanning electron microscope (JEOLJSM–6380LV).

### Extant material and preparation

The leaves of extant *Platycladus orientalis* for comparison were collected from Lanzhou Botanical Garden (36°07'08"N, 103°42'08"E), China. Other relevant extant species of the Cupressaceae were collected from Kunming Botanical Garden (25°05'05"N, 102°46'34"E), Shanghai Botanical Garden (31°08'54"N, 103°42'18"E) and Beijing Botanical Garden (39°59'56"N, 116°12'49"E), China. The cuticles of extant leaves were prepared following the method described by Wu et al. [14].

All specimens and cuticle slides are housed in the Institute of Paleontology and Stratigraphy, Lanzhou University, China. Terminology on leaf morphology follows Fu et al. [1] and Farjon [2], while terms on foliar cuticle are adopted after Kvaček et al. [7] and Shi et al. [9], [15], [16].

### Nomenclature

The electronic version of this article in Portable Document Format (PDF) in a work with an ISSN or ISBN will represent a published work according to the International Code of Nomenclature for algae, fungi, and plants, and hence the new names contained in the electronic publication of a PLOS ONE article are effectively published under that Code from the electronic edition alone, so there is no longer any need to provide printed copies. The online version of this work is archived and available from the following digital repositories: PubMed Central, LOCKSS.

### Ethics Statement

All necessary permits were obtained for the described sampling sites in verbal or written form. Eight fossils of *Platycladus* were collected in Lianghe County, Yunnan Province, China. The field work is permitted by the local government. For the extant plant sampling sites, permits were obtained from the botanical garden offices in verbal. The extant plant materials did not involve endangered or protected species.

## Results

**Family.** Cupressaceae Gray *sensu lato*

**Genus.** *Platycladus* Spach

**Species.** *Platycladus yunnanensis* J.Y. Wu, sp. nov.

**Figure 3:**
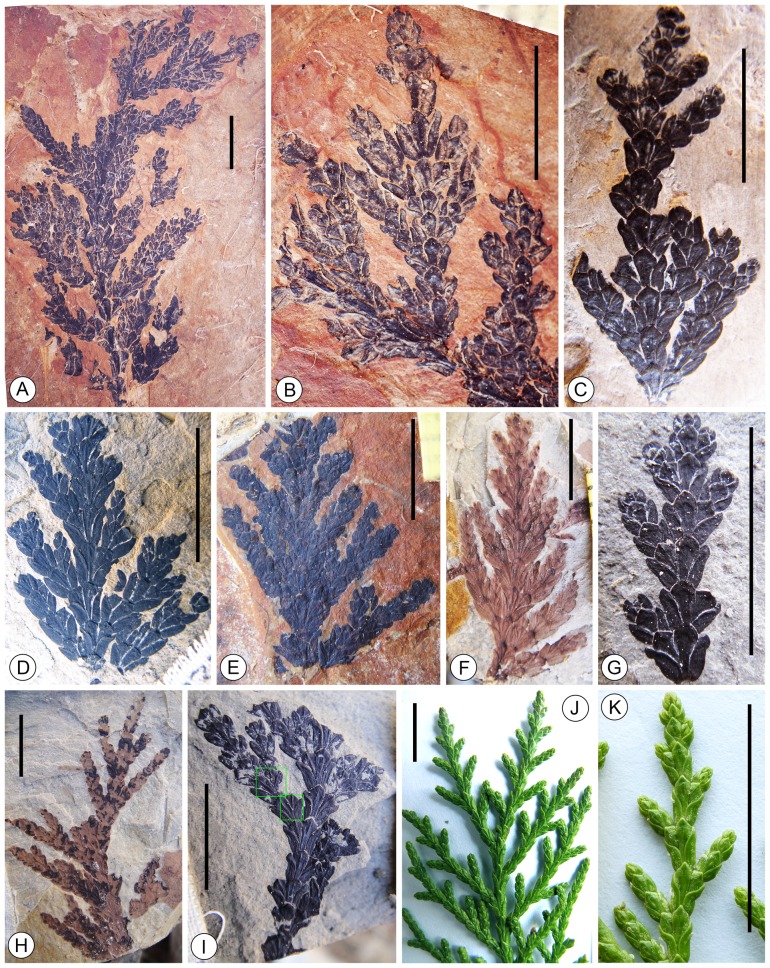
Foliage branches of *Platycladus yunnanensis* sp. nov. and extant *Platycladus orientalis*. Scale bar  = 1 cm. A-I. *Platycladus yunnanensis* sp. nov. A, B. Holotype. Specimen no. LZLH–3021. C. Specimen no. LULH–4487. D. Specimen no. LULH–4335. E. Specimen no. LZLH–3312. F. Specimen no. LULH–4436. G. Specimen no. LULH–3344. H. Specimen no. LULH–4423. I. Specimen no. LULH–3349. Green box shows the leaves in the bifurcation of branchlets. J, K. Extant *Platycladus orientalis*.

**Figure 4:**
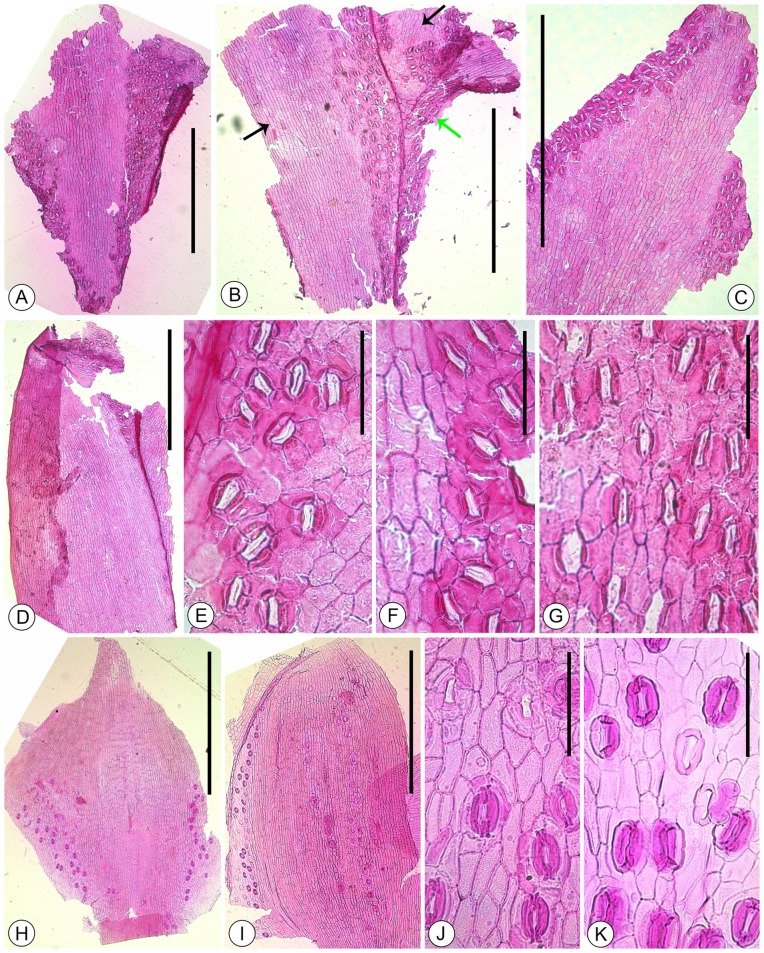
A-G. Cuticles of *Platycladus yunnanensis* sp. nov. under light microscope. A. Abaxial cuticle of facial leaf. LZLH–3021. B. Cuticles of the facial leaves (black arrows) and lateral leaf (green arrow) in the bifurcation of branchlets. Sampling from the area of green box in Fig. 3I. C. Abaxial cuticle of lateral leaf. LULH–4487. D. Adaxial cuticle of lateral leaf. LULH–4487. E. Stomatal zone of the abaxial cuticle of facial leaf. LZLH–3021. F. Stomatal zone of the adaxial cuticle of facial leaf. LZLH–3021. G. Stomatal zone of the abaxial cuticle of lateral leaf. LZLH–3021. H-K. Cuticles of extant *Platycladus orientalis* under light microscope. H. Adaxial cuticle of facial leaf. I. Adaxial cuticle of lateral leaf. J. Stomatal zone in the abaxial cuticle of facial leaf. K. Stomatal zone in the abaxial cuticle of lateral leaf. A-D, H, I. Scale bar  = 1 mm. E-G, J, K. Scale bar  = 100 µm.

**Figure 5:**
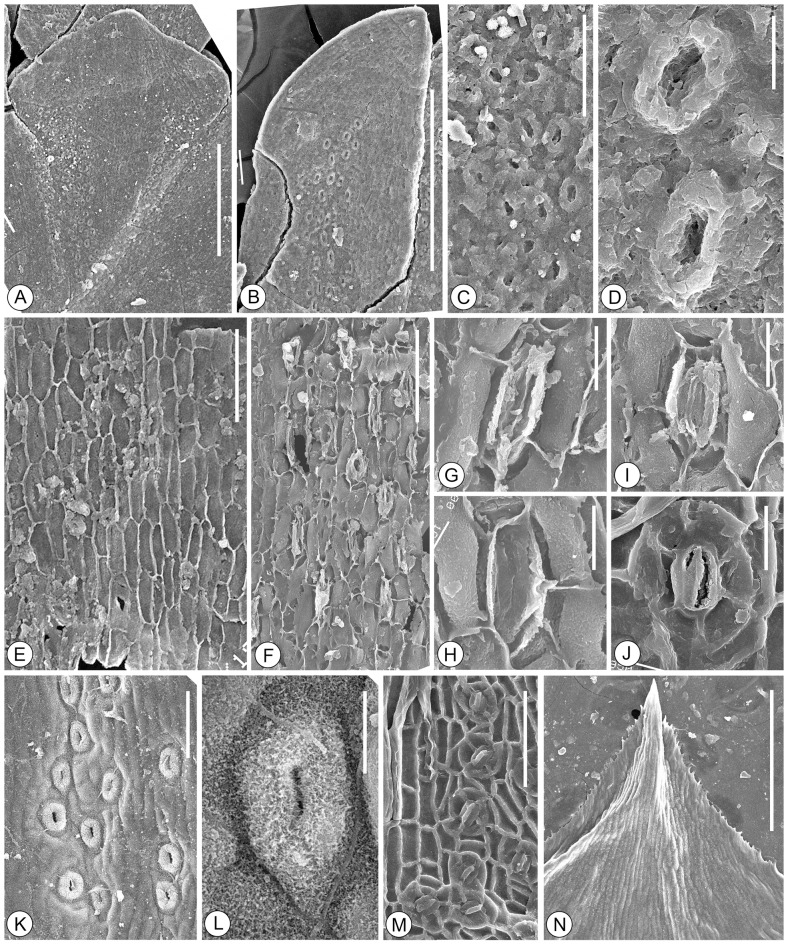
A-I. Cuticles of *Platycladus yunnanensis* sp. nov. under SEM. A. Outer view of the facial leaf, showing the entire margin. LZLH–3021. B. Outer view of the lateral leaf. LZLH–3021. C. Outer view of the stomatal zone in the abaxial cuticle of facial leaf, showing the Florin rings. LZLH–3021. D. Outer view of the stomata in the abaxial cuticle of lateral leaf, showing the elliptical stomatal pit and surrounding Florin ring. LZLH–3021. E. Inner view of the epidermal cells in the nonstomatal zones of facial leaf. LULH–4335. F. Inner view of the stomatal zone in the abaxial cuticle of lateral leaf. LULH–4335. G-H. Inner views of stomatal complexes, showing 5–7 subsidiary cells with smooth periclinal walls. J-M. Cuticles of extant *Platycladus orientalis* under SEM. J. Inner views of a stomatal complex. K. Outer view of the stomatal zone in lateral leaf. L. Outer view of a stoma in the abaxial cuticle of lateral leaf. M. Inner view of the stomatal zone in the abaxial cuticle of facial leaf. N. Cuticle of extant *Calocedrus macrolepis* under SEM, showing the minutely serrate margins. A, B, N. Scale bar  = 500 µm. C, E, F, K, M. Scale bar  = 100 µm. D, G-J, L. Scale bar  = 20 µm.

**Figs.** 3A–I ; 4A–G ; 5A–I

**Holotype.** Specimen no. LZLH–3021 (designated here).

**Paratypes.** Specimen nos. LULH–4487, LULH–4335, LZLH–3312, LULH–4436, LULH–3344, LULH–4423, LULH–3349 (designated here).

**Horizon.** Nanlin Formation.

**Locality.** Nongbie Village (24°51'46"N, 98°24'58"E), Lianghe County, Yunnan Province, China.

**Age.** Late Miocene.

**Number of specimens studied.** Eight.

### Diagnosis

Foliage branches spreading in flattened sprays. Leaves decussate, imbricate, scale–like, dimorphic. Facial leaves rhombic to obtrullate, with an obtuse apex and entire margins.. Lateral leaves bilaterally flattened, boat–shaped, with an obtuse apex and entire margins. Leaves amphistomatic. Epidermal cells rectangular or with oblique end walls. Periclinal walls smooth in the inner surface and rough in the outer surface, anticlinal walls straight. Stomata in irregular rows, fewer on the adaxial cuticle. Stomata elliptical or oblong, haplocheilic, monocyclic. Guard cells slightly sunken and encircled by 5–7 subsidiary cells. Stomatal pits elliptical, open and shallow. Each stoma surrounded by a distinct Florin ring on outer cuticle surface.

### Description

Foliage branches spreading in flattened sprays (Fig. 3A–I). Ultimate branchs arise from the axils of the lateral leaves of penultimate branchs. Leaves are decussate, imbricate, scale-like, dimorphic in facial and lateral leaves. The leaves are sessile, with the base decurrent. The facial leaves are similar in size or slightly smaller than the laterals (Fig. 3B, C, D, F, G). Facial leaves are rhombic to obtrullate, 2.0–3.0 mm (mean 2.5 mm) long and 1.2–2.0 mm (mean 1.5 mm) wide, with a median groove abaxially (Fig. 3C, G); the apex is appressed and obtuse, and the leaf margins is entire (Fig. 3C, G; 5A). Lateral leaves are conduplicate, bilaterally flattened, boat–shape, the distal part spreading or reflexed, 2.2–3.2 mm (mean 2.7 mm) long and 1.2–1.8 mm (mean 1.4 mm) wide; the apex is appressed or free, incurved and obtuse, and the leaf margins is entire (Fig. 3C, G; 5B).

The lateral leaves are amphistomatic. The abaxial cuticle has two stomatal zones arranged on each side of the upper part (Fig. 4C). In the median nonstomatal zones, the epidermal cells are rectangular or with oblique end walls, usually elongate along the leaf long axis, 35–100 µm long and 15–30 µm wide, with a length to width ratio up to 5. The anticlinal walls are straight (Fig. 4C; 5E). The periclinal walls are almost smooth on the inner surface and rough on the outer surface (Fig. 5B, E). The stomatal zone often contains 4–7 irregular rows of stomata along the leaf long axis. The epidermal cells in the stomatal zones are irregular. The stomata are irregularly arranged, oriented longitudinally or orientation (Fig. 4G). The adaxial cuticle has fewer stomata. In the nonstomatal zones, the epidermal cells are rectangular or with oblique end walls, strongly elongate along the leaf long axis, strongly elongate, 48–110 µm long and 10–22 µm wide, with a length to width ratio up to 9. The stomatal zone is arranged on each side of the upper part along the leaf long axis, often containing two rows of stomata (Fig. 4D).

The facial leaves are amphistomatic. The abaxial cuticle is composed of a median nonstomatal zone and two broad stomatal zones on each side. In the nonstomatal zones, the epidermal cells are rectangular or with oblique end walls, more or less elongate, 30–80 µm long and 15–30 µm wide, with a length to width ratio up to 6. The anticlinal walls are straight or sometimes curved, the periclinal walls are smooth in the inner surface and rough on the outer surface. The stomatal zone is V–shaped, with 6–8 lines of stomata in the upper part, and decreases downward (Fig. 4A; 5F). The stomata are irregularly arranged, mostly oriented longitudinally parallel to the long axis of the leaf, or oriented with a little deflexion (Fig. 4E, F). The epidermal cells in the stomatal zones are irregular. The adaxial cuticle has fewer stomata, with 2–3 lines of stomata on the upper part along the leaf long axis.

The lateral and facial leaves have similar stomatal complexes. The stomata complexes are elliptical or oblong, 30–48 µm long and 20–32 µm wide, haplocheilic, monocyclic (Fig. 5G–J). The guard cells are encircled by 5–7 subsidiary cells. The stomatal pits are usually open and shallow, elongate, elliptical in outline, 12–20 µm long and 4–9 µm wide (Fig. 5C, D). The guard cells are slightly sunken, usually forming a closed aperture, with periclinal walls inner cuticle surface almost smooth under the SEM. The subsidiary cells are usually two polar cells and the others lateral, quadrangular. The periclinal walls of subsidiary cells are usually smooth in the inner surface. The subsidiary cells are cambered outwards in the outer surface and form a distinct Florin ring around the stomatal pit (Fig. 5C, D). The Florin ring usually is elliptical in outline, 22–30 µm long and 14–25 µm wide, with a thickened, lobed rim 6–8 µm wide.

### Affinities

The present fossil branches are spreading in flattened sprays, and possess decussate, imbricate and scale–like leaves. It is certain that the gross morphology of the present fossils are of the family Cupressaceae *s.l.* In the Cupressaceae (Table 1), the genera *Cuninghamia* R.Br., *Taiwania* Hayata, *Athrotaxis* D. Don, *Sequoiadendron* Buchholz, *Sequoia* Endl., *Cryptomeria* D. Don, *Taxodium* L. and *Glyptostrobus* Endl. have leaves that are helically inserted, differ from our fossils that have leaves arranged decussately [2]. The foliage branches of *Cupressus* L. and *Widdringtonia* Endl. often spread in a nonplanar arrangement [16]. The leaves of *Callitris* Vent., *Actinostrobus* Miq. and *Fitzroya* are arranged in whorls of 3 or 4. *Juniperus* differs from the present fossils in the absence of dimorphic leaves [2], [17]. The foliage branches of *Austrocedrus* Florin et Boutelje, *Diselma* Hook. f., *Pigerodendron* Florin and *Tetraclinis* are also spreading not in a plane (Table 1).

**Table 1:** Comparison of foliage morphology and stomatal distribution with the genera of Cupressoideae *s.l.* [1], [2], [7], [15].

Genus	Foliage branches	Leaf arranged	Leaf shape	Stomatal distribution
*Platycladus yunnanensis* sp. nov.	In flattened sprays	Decussate, imbricate	Dimorphic	Amphistomatic
*Cuninghamia*	Opposite	Helically	narrowly lanceolate or linear-lanceolate	Amphistomatic
*Taiwania*	Alternate	Alternate to helically	falcate-subulate	Amphistomatic
*Athrotaxis*	Forming a conical crown	Helically	rhombic-ovate to linear-lanceolate	Amphistomatic
*Sequoiadendron*	Alternate	Helically	Homomorph; variable of shapes	Amphistomatic
*Sequoia*	Horizontally and flattened	Alternate or near helically	Heteromorph, linear	Hypostomatic
*Metasequoia*	Opposite	Opposite	Homomorph; linear	Hypostomatic
*Cryptomeria*	Dense	Helically	Homomorph; linear-subulate	Amphistomatic
*Taxodium*	Dimorphic, sympodial or alternate	Helically (or pectinately)	Linear or acicular	Amphistomatic
*Glyptostrobus*	Alternate	Alternate to helically	scale-like or lanceolate	Amphistomatic
*Thujopsis*	In flattened sprays	Decussate, imbricate	Dimorphic	Amphistomatic
*Thuja*	In flattened sprays	Decussate, imbricate	Dimorphic	Amphistomatic
*Fokienia*	In flattened sprays	Decussate, imbricate	Dimorphic	Amphistomatic
*Chamaecyparis*	In flattened sprays	Decussate, imbricate	Dimorphic	Amphistomatic
*Cupressus*	Often decussately arranged (not in a plane)	Decussate, imbricate	Monomorphic or dimorphic	Amphistomatic
*Juniperus*	Irregularly disposed (not in a plane)	in whorls of 3 or decussate	Not dimorphic	Amphistomatic
*Calocedrus*	In flattened sprays	Decussate, imbricate	Dimorphic	Amphistomatic
*Tetraclinis*	Articulate, alternate at various angles (not in a plane)	Decussate	Weakly dimorphic	Amphistomatic
*Platycladus*	In flattened sprays	Decussate, imbricate	Dimorphic	Amphistomatic
*Microbiata*	In flattened sprays	Decussate, imbricate	Weakly dimorphic	Amphistomatic
*Xanthocyparis*	In flattened sprays	Decussate or in whorls of 4	Dimorphic or monomorphic	Amphistomatic
*Papuacedrus*	In flattened sprays	Decussate or in whorls of 4	Strongly dimorphic	Amphistomatic
*Libocedrus*	Frondose, forming dense sprays; or in flattened sprays (*Libocedrus bidwillii*)	Decussate, imbricate	Dimorphic or nearly monomorphic	Amphistomatic
*Pigerodendron*	Irregularly disposed (not in a plane)	Decussate, imbricate	Lanceolate	Epistomatic
*Austrocedrus*	Dense	Decussate	Dimorphic	Amphistomatic
*Diselma*	Dense, not in a plane	Opposite-decussate	Monomorphic, rhombic	Hypostomatic
*Fitzroya*	Not in a plane	In alternate near-whorls of 3	Lanceolate to ovate	Amphistomatic
*Widdringtonia*	Spreading erect (not in a plane)	Decussate or spirally	Ovate to rhombic	Amphistomatic
*Neocallitropsis*	Dense tufts	In whorls of 4	lanceolate	Amphistomatic
*Actinostrobus*	Irregularly disposed (not in a plane)	In whorls of 3	Linear-lanceolate	Amphistomatic
*Callitris*	In tufts	In whorls of 3	Linear	Epistomatic

**Figure 6:**
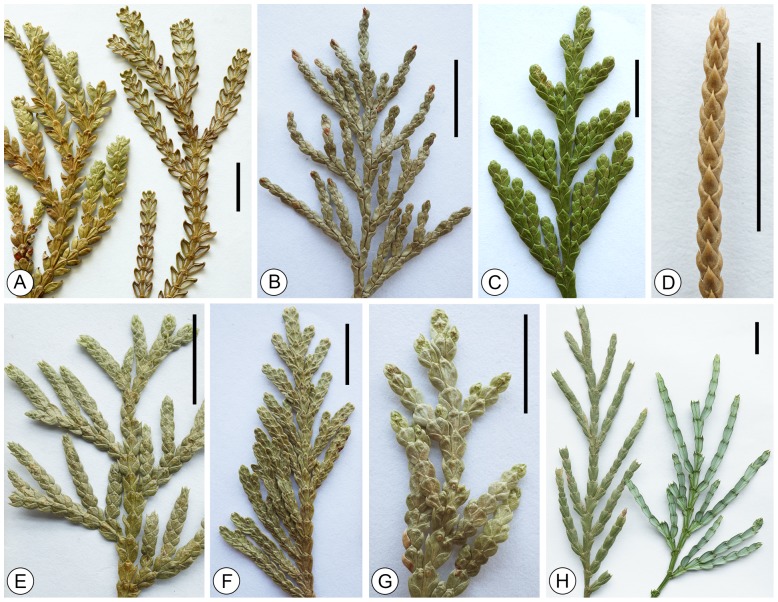
Foliage branches of extant species of Cupressaceae for comparison with *Platycladus yunnanensis* sp. nov. Scale bar  = 1 cm. A. *Thujopsis dolabrata*. B. *Chamaecyparis obtuse.* C. *Thuja occidentalis*. D. *Microbiata decussate.* E. *Chamaecyparis pisifera.* F. *Thuja plicata*. G. *Thuja standishii.* H. *Calocedrus macrolepis.*

Our fossil branches distinctly spread in a plane and the leaves are amphistomatic, a distinction which only occurs in the genera *Thujopsis* Sieb. et Zucc. ex Endl., *Thuja* L., *Fokienia* A. Henry et H.H. Thomas, *Chamaecyparis* Spach, *Calocedrus* Kurz, *Platycladus, Microbiata* Kom., *Xanthocyparis* Farjon et Hiep, *Papuacedrus* H.L. Li and *Libocedrus* Endl. (Table 1). However, most species of these genera are different from our fossils in leaf morphology (Table 2). For example, *Thujopsis dolabrata* (Thunb. ex L. f.) Sieb. et Zucc. has obovate–obdeltoid facial leaves with serrate margins [9], and its lateral leaves are dolabriform with a distinctly median groove of stomatal zone (Fig. 6A). *Thuja sutchuenensis* Franch. and *T. koraiensis* Nakai have broadly falcate lateral leaves (Table 2), *T. standishii* (Gordon) Carrière possesses glands on the facial leaves (Fig. 6G), *T. occidentalis* L. and *T. plicata* Donn ex D. Don have ultimate branchlets often more numerous on the acroscopic side of lateral branchlets (Fig. 6C, F; [2]). *Fokienia hodginsii* (Dunn) A. Henry et H.H. Thomas possess a larger leaf sizes than our fossils [2], and the papillae around the stomata in *F. hodginsii* [18] are absent in the present fossils. The species within *Chamaecyparis* Spach possess rhombic to lanceolate facial leaves and broadly falcate to lanceolate lateral leaves, usually with acute apices (Fig. 6B, E). *Xanthocyparis nootkatensis* (D. Don) Farjon et Harder possesses ultimate branchlets often unilateral on the second highest order, and *X. vietnamensis* Farjon et Hiep has broadly falcate to lanceolate laterals with minutely serrate margins [2]. *Papuacedrus papuana* (F. Muell.) H.L. Li has much smaller facials than the laterals. *Libocedrus bidwillii* Hook. f. has acute apices in the facial and lateral leaves. *Calocedrus* species possess oblong to obtrullate facial leaves with serrate margins (Fig. 5N), and linear–lanceolate laterals with acute apices (Fig. 6H). Therefore, we can conclude that all the species in Cupressoideae, except for *Platycladus orientalis*, are differ from the present fossils (Table 2). However, some minor differences can also be found between *P. orientalis* and our fossil branches, such as *P. orientalis* possessing smaller leaves (1.5–2 mm long and 1–1.5 mm wide) [2] than those in our fossils (2–3.2 mm long and 1.2–2.0 mm wide), and the stomata of our fossils being more or less elongate (Fig. 5G–J).

**Table 2:** Comparison of leaf morphology with the relevant species of Cupressoideae *s.l.* [1], [2].

Species	Facial shape	Facial apex	Lateral shape	Lateral apex	Facials vs. laterals
*Platycladus yunnanensis* sp. nov.	Rhombic to obtrullate	Obtuse	Bilaterally flattened	Incurved, obtuse	Slightly smaller
*Thujopsis dolabrata*	Obovate to obdeltoid	Obtuse	Dolabriform	Incurved, obtuse or acute	Similar
*Thuja sutchuenensis*	Rhombic to obtrullate	Obtuse	Broadly falcate	Incurved, obtuse	Slightly smaller
*Thuja koraiensis*	Obtrullate to rhombic	Obtuse	Broadly falcate	Incurved, obtuse or acute	Smaller
*Thuja standishii*	Rhombic to obtrullate	Obtuse or acute	Bilaterally flattened	Incurved, acute-apiculate	Slightly smaller
*Thuja occidentalis*	Rhombic to obtrullate	Obtuse	Bilaterally flattened	Acute or obtuse	Slightly smaller
*Thuja plicata*	Rhombic to obtrullate	Obtuse	Bilaterally flattened	Incurved, acute	Equally long or slightly smaller
*Fokienia hodginsii*	Oblanceolate or variable	Obtuse or acuminate	Bilaterally flattened	Acute to obtuse	Shorter or nearly equal
*Chamaecyparis thyoides*	Rhombic to ovate-oblong	Obtuse or acuminate	Broadly falcate to lanceolate	Incurved at the appressed apex	Slightly smaller
*Chamaecyparis pisifera*	Rhombic to obovate	Obtuse to acuminate	Broadly falcate	Incurved at the appressed apex	Slightly smaller
*Chamaecyparis lawsoniana*	Rhombic to lanceolate	Obtuse to acuminate	Broadly falcate to lanceolate	Incurved at the appressed apex	Slightly smaller
*Chamaecyparis formosensis*	Rhombic to lanceolate	Obtuse to acuminate	Lanceolate	Incurved, acute	Slightly smaller
*Chamaecyparis obtusa*	Rhombic to oblong	Obtuse	Broadly falcate to lanceolate	Incurved, obtuse	Slightly smaller
*Calocedrus decurrens*	Oblong to obtrullate	Obtuse to acuminate	Linear-lanceolate	Incurved, acute	Slightly smaller
*Calocedrus formosana*	Oblong to obtrullate	Obtuse to acuminate	Linear-lanceolate	Incurved, acute	Slightly smaller
*Calocedrus macrolepis*	Oblong to obtrullate	Obtuse to acuminate	Linear-lanceolate	Incurved, acute	Slightly smaller
*Platycladus orientalis*	Rhombic to obtrullate	Obtuse	Bilaterally flattened	Incurved, obtuse	Slightly smaller
*Microbiata decussata*	Rhombic	Narrower acuminate or acute	Rhombic	Narrower acuminate or acute	Smaller
*Xanthocyparis nootkatensis*	Narrowly rhombic to lanceolate	Acuminate to acute	Broadly falcate to lanceolate	Incurved, acute	Similar
*Xanthocyparis vietnamensis*	Narrowly ovate-rhombic	Acute to acuminate	Straight or falcate	Acute or pungent	Slightly shorter
*Papuacedrus papuana*	Rhombic to lanceolate	Cuspidate	Bilaterally flattened	Incurved, obtuse or acute	Much smaller
*Libocedrus bidwillii*	Rhombic	Apiculate to acute	Bilaterally flattened	Apiculate to acute	Smaller

Zhang [12] reported some cupressaceous fossils as *Calocedrus lantenoisii* (Laurent) Tao from the Miocene Nanlin Formation, but without any figure and description. This fossil species has been widely reported from the Oligocene to the Miocene in Yunnan [12], [19], [20], and resembles the extant *C. macrolepis* Kurz in gross morphology [20]. Some fossil foliage shoots from the Oligocene in Guangxi, South China have been described as *C. huashanensis* [9]. The lateral leaves of *C. huashanensis* are falcate with acute to acuminate apices. In any case, the leaves with serrate and scariose margins in the extant and fossil *Calocedrus* [9] distinctly differ from those of our fossils. *Fokienia shengxianensis* He, Sun et Liu from the Miocene of Zhejiang, East China [18] has a leaf shape similar to that of our fossils. However, the leaves of *F. shengxianensis* are hypostomatic and the stomata are encircled by many papillae. Based on the comparisons above, all the extant species and previously fossil species of Cupressaceae are more or less different from our fossils, which supports their designation as a new fossil species of *Platycladus*.

## Discussion

**Figure 7:**
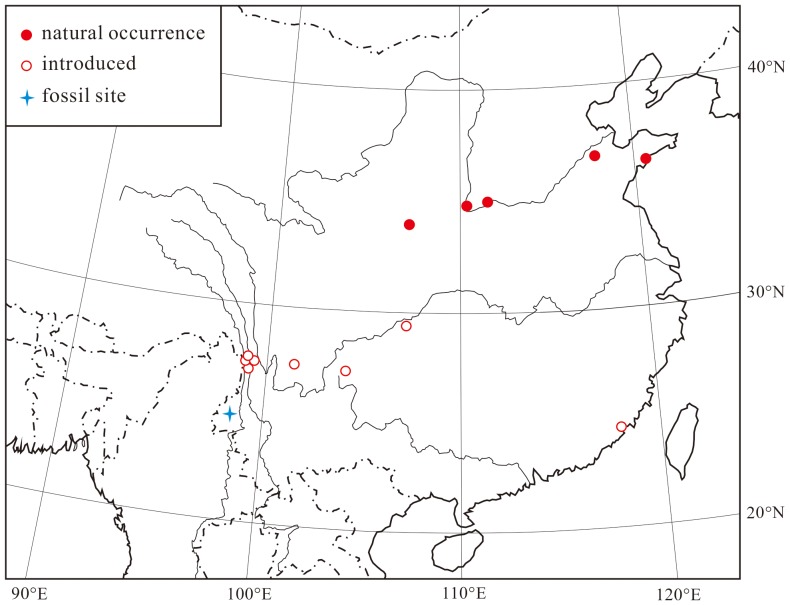
Distribution of extant *Platycladus orientalis* and fossil site of *Platycladus yunnanensis* sp. nov. [2].

The genus *Platycladus* only contains one extant species, *P. orientalis*, native to S Gansu, Hebei, Shaanxi and Shanxi of China, and introduced or status uncertain in Korea and the Russian Far East [1], [2]. It is therefore very difficult to establish its original natural range [1], [2]. Wilson [21] argued that the species occurs naturally in the deep valleys of the Jinshan, Lancang, and Nu River of Northwest Yunnan and Southwest Sichuan, China. However, Farjon [2] is certain that the occurrences in Yunnan and Sichuan are not indigenous, and there had been no evidence in the fossil record to indicate a historical natural distribution more southernly than at present. In the present study, the occurrence of *P. yunnanensis* sp. nov. in western Yunnan suggests that this genus might have a more southernly natural distribution during the late Miocene, which is beyond the bounds of the modern natural distribution of this genus (Fig. 7) if Farjon's opinion is correct.

The climate of the natural range of *Platycladus orientalis* is characterized by relatively dry and moderately cold winter conditions [2]. However, *P. orientalis* is introduced extensively in China due to its toleration of a wide range of climate and soil conditions [3], [22]. The present fossil leaves have open stomatal pits and shallowly sunken guard cells, usually reflecting a humid climate [9]. Therefore, the late Miocene *P. yunnanensis* sp. nov. should also grow under a humid subtropical climate in West Yunnan. Such a climate inference is supported by previous quantitative analyses of several late Miocene floras in southwestern China [23]–[25].

On the basis of molecular phylogenetic studies within the Cupressaceae *s.l.*, two Asian species, *Platycladus orientalis* and *Microbiota decussate*, form a clade [4], [5]. Mao et al. [4] suggested that the age of the node of *Platycladus*–*Microbiota* is ca. 33 Ma (Early Oligocene). However, the earliest fossils of *Platycladus* and *Microbiota* [10] only date back to the late Miocene and Pliocene, respectively. Phylogenetic data indicate that the clade *Platycladus*–*Microbiota* is closely related to the genera *Tetraclinis* and *Calocedrus* [4], [5]. The earliest fossils of *Tetraclinis* [7] and *Calocedrus* [9] are documented from the Oligocene. However, Kvaček et al. [7] indicated that the genus *Tetraclinis* had migrated between western North America and Europe during the Oligocene or Miocene through the North Atlantic land bridge, but did not cross Asia. Brunsfeld et al. [26] indicated that the clade *Platycladus*–*Microbiota* is close to *Calocedrus*, but *Tetraclinis* is the sister taxon to *Thuja* and *Thujopsis* based on the *rbc*L sequences.

The extant eastern Asian *Calocedrus* usually occurs in mixed evergreen conifer–broadleaved forests in the subtropical or tropical montane areas [27]. However, extant *Platycladus* and *Microbiota* are found in a climate of relatively dry and very cold winters [2]. The molecular phylogenetic studies indicate that the divergence between *Platycladus* and *Microbiota*, as well as the species among *Calocedrus* both happened during the Oligocene [4], [8]. If the molecular phylogenetic evidence proved to be correct, the separation between the clade *Platycladus*–*Microbiota* and the genus *Calocedrus* was most likely a result of the broad arid belt across Central China during the Oligocene [28]–[30]. In addition, the the cooling down of the global temperature [31] and the strengthening of Asian monsoon [32]–[37] since the middle Miocene further promoted the migration of these genera. The deep split between eastern Asian and North American *Calocedrus* before the Oligocene via the Bering land bridge has been demonstrated [8], [9]. However, due to the inadequate paleobotanical data, the differentiation of *Platycladus* and *Microbiota* is not well defined.
